# Variational Channel Estimation with Tempering: An Artificial Intelligence Algorithm for Wireless Intelligent Networks

**DOI:** 10.3390/s20205939

**Published:** 2020-10-21

**Authors:** Jia Liu, Mingchu Li, Yuanfang Chen, Sardar M. N. Islam, Noel Crespi

**Affiliations:** 1 School of Software Technology and Key Laboratory for Ubiquitous Network and Service Software, Dalian University of Technology, Dalian 116620, China; jialiudlut@gmail.com (J.L.); mingchul@dlut.edu.cn (M.L.); 2 School of Cyberspace, Hangzhou Dianzi University, Hangzhou 310018, China; 3 Institute for Sustainable Industries and Liveable Cities, Victoria University, Melbourne 14428, Australia; Sardar.Islam@vu.edu.au; 4 Institut Polytechnique de Paris, Institut Mines-Telecom, Telecom SudParis, 91011 Evry CEDEX, France; noel.crespi@mines-telecom.fr

**Keywords:** artificial intelligence algorithm, truth inference, channel estimation, message passing

## Abstract

With the rapid development of wireless sensor networks (WSNs) technology, a growing number of applications and services need to acquire the states of channels or sensors, especially in order to use these states for monitoring, object tracking, motion detection, etc. A critical issue in WSNs is the ability to estimate the source parameters from the readings of a distributed sensor network. Although there are several studies on channel estimation (CE) algorithms, existing algorithms are all flawed with their high complexity, inability to scale, inability to ensure the convergence to a local optimum, low speed of convergence, etc. In this work, we turn to variational inference (VI) with tempering to solve the channel estimation problem due to its ability to reduce complexity, ability to generalize and scale, and guarantee of local optimum. To the best of our knowledge we are the first to use VI with tempering for advanced channel estimation. The parameters that we consider in the channel estimation problem include pilot signal and channel coefficients, assuming there is orthogonal access between different sensors (or users) and the data fusion center (or receiving center). By formulating the channel estimation problem into a probabilistic graphical model, the proposed Channel Estimation Variational Tempering Inference (CEVTI) approach can estimate the channel coefficient and the transmitted signal in a low-complexity manner while guaranteeing convergence. CEVTI can find out the optimal hyper-parameters of channels with fast convergence rate, and can be applied to the case of code division multiple access (CDMA) and uplink massive multi-input-multi-output (MIMO) easily. Simulations show that CEVTI has higher accuracy than state-of-the-art algorithms under different noise variance and signal-to-noise ratio. Furthermore, the results show that the more parameters are considered in each iteration, the faster the convergence rate and the lower the non-degenerate bit error rate with CEVTI. Analysis shows that CEVTI has satisfying computational complexity, and guarantees a better local optimum. Therefore, the main contribution of the paper is the development of a new efficient, simple and reliable algorithm for channel estimation in WSNs.

## 1. Introduction

### 1.1. Problem Statement

The recent growth of the mobile Internet has brought a tremendous increase in the number and types of data services, creating much more challenging requirements for 5G communications. Meanwhile, the development of the 5G network relies highly on the improvement of WSNs, which are comprised of a large number of cheap devices that can be used to observe and preprocess neighboring physical and environmental changes. To construct an efficient path through the tremendous amount of base stations, millimeter wave technology is one of the critical parts of 5G technology [1,2,3]. Therefore, for 5G networks, it is essential to know the channel impulse response (CIR) of the millimeter waves, i.e., to estimate the channel pattern between different base stations. Luckily, the technological breakthrough of micro-electro-mechanical systems (MEMS) has promoted the application of large scale WSNs [4,5,6]. Thanks to the advantageous properties of self-organization and real-time data preprocessing, WSNs are a promising technology that can contribute to future 5G communications [7,8,9]. However, there are some challenges underlying this realization, since it is still difficult to deploy certain sensor networks to execute specific tasks. One of the challenges is limited energy resources, as it is energy-consuming to deploy many sensors awaiting task assignment and computing duties. It is, therefore, imperative to develop energy efficient algorithms in WSNs.

A central problem in WSNs technology is its use of data collected by a network of spatially distributed sensors to estimate channel parameters. Sensors can cooperate to send their individual raw or preprocessed data to a data fusion center (FC) when requested. The FC then makes use of the collected data to extract the desired information. This process differs from the traditional centralized system, given that in a centralized system, all data are available and can be accessed in real time. Since the centralized estimation problem is no longer an issue, the existing works are all considered to be decentralized estimation.

### 1.2. Previous Research and Limitations

The issue of channel estimation has attracted broad interest in the past few years, and the existing algorithms can be classified as least square (LS)/minimum mean square error (MMSE)-based, compressive sensing (CS)-based, message passing (MP)-based, deep learning (DL)-based, or VI-based [10,11,12].

Conventional channel estimation algorithms typically include LS, MMSE, or expectation maximization (EM)-based methods. In 2005, Qian et al. [13] researched into iterative LS-based channel estimation algorithms, and showed that LS works well in mobile networks. In 2010, Simko et al. [14] proposed an approximate linear minimum mean square error (ALMMSE) fast fading CE algorithm. In 2012, Wang et al. [15] proposed a soft-output MMSE channel estimation algorithm under correlated Rician fading MIMO channels. In 2013, Zhang et al. [16] proposed a distributed angle estimation (or channel estimation) algorithm based on space-alternating generalized expectation maximization. Compared with the LS method, MMSE, including linear minimum mean square error (LMMSE, [17]), considers noise and therefore improves the accuracy. However, with matrix inverse computations, LMMSE has higher computation complexity. Therefore, the conventional channel estimation algorithms (LS, MMSE, etc.) are obviously flawed with their problems of high complexity and inability to scale, which can rapidly lead to failure in massive MIMO scenarios. In a massive MIMO, as the number of antennas in each base station (BS) increases, the downlink pilot training overhead and uplink channel state information (CSI) feedback overhead can quickly become overwhelming. Therefore, the pilots for evaluating CSI are inadequate.

A CS-based approach can be used to solve the above issue with conventional channel estimation algorithms. From the simulated study we can see that limited by the number of scatterers in the BS, the user channel matrix tends to be sparse in massive MIMO scenarios. In this case, it is ineffective to use long training symbols to estimate CSI. CS can mitigate this disadvantage and make use of user channel matrix sparsity. In 2014, Rao et al. [18] proposed a distributed compressive transmitter side channel state information (CSIT) estimation scheme. In their work, they locally observed the compressed measurements while recovering the CSIT at the base station. In 2016, Tseng et al. [19] proposed a new CS-based downlink CSI estimation scheme for frequency division duplexing (FDD) massive MIMO systems (CE for FDD). However, there are still challenges in CS-based channel estimation algorithms, such as the difficulty to exploit joint channel sparsity among multiple users, and to decide how sparsity can affect performance. Although some researchers have tried to analyze this issue, in general, these are specific analyses, and thus cannot be extended.

Owing to its convergence guarantee property and distributed nature, the MP algorithm is also used to implement channel estimation. In 2005, Paskin et al. [20] proposed a robust distributed sensor network inference architecture and exploited MP to compute the global parameter after forming a spanning tree and a junction tree. In 2019, Bellili et al. [21] proposed a generalized approximate message passing (GAMP) algorithm to find the ground truth of the mmWave MIMO channel coefficient matrix. However, MP-based CE algorithms are too specific to generalize in different types of networks.

Some researchers use DL to estimate channel due to its strong computing power. In 2017, Ye et al. [22] proposed a DL-based CE algorithm to train a deep neural network (DNN) under different channel conditions, and then applied it online to recover the transmitted data. In 2018, He et al. [23] proposed a DL-based channel estimation for beam-space mmWave massive MIMO systems. It is interesting that the authors also use a learned denoising-based approximate message passing network (LDAMP), i.e., they combined the use of DL and MP. In 2020, Zhang et al. [24] proposed a deep reinforcement learning algorithm for double coded caching. In summary, DL-based CE algorithms use deep neural networks to learn the channel feature, and obtain the complete channel information from received pilot symbols. Some DL-based CE models treat channel information as a picture and use the CS-based method to solve channel state information. Despite the high accuracy of DL-based CE algorithms, it is impractical to obtain labeled data or train self-adaptive neural networks for channel estimation. Therefore, it is still difficult to develop DL-based CE algorithms that have real-time generality and efficiency properties.

Some researchers try to use statistical methods to solve the CE problem by forming it into a statistical optimization problem. Treating the channel state information as a set of samples generated by a distribution, we can write the joint posterior density to be maximized as the objective function. However, in statistical inference, it is often too difficult to directly optimize the objective function when computing joint posterior density or other statistical variables. Therefore, we opt for an alternative suboptimal approach, which is VI. In VI, we use another distribution that has minimal KL divergence, or variational free energy, or evidence lower bound (ELBO), with the approximated distribution. By approximating the difficult-to-compute distribution, we can guarantee faster convergence and optimum performance (see Ahad et al. [25]). Please note that many studies that use MP, MF and BP are categorized as VI, and use factor graphs as their graphical models. We classify MP-based channel estimation (CE) algorithms as a specific group, because unlike MF and BP, MP is the direct model for autonomous systems that communicate two-way. In 2008, Hu et al. [26] proposed a divergence minimization approach to derive iterative decoding algorithms in coded CDMA systems. They stated that the KL divergence is equivalent to the variational free energy, both of which can be used to measure the distribution distance between true distribution and auxiliary distribution. In 2010, Kirkelund et al. [27] proposed a variational message passing (VMP) architecture that can estimate the channel coefficients and inverse noise variance matrix in a semi-blind way. As with Hu et al. [26], this scheme is also an iterative decoding channel estimation approach, which only differs in proposing the notion of VI. In 2012, Ahmad et al. [25] proposed a simpler suboptimal approach that uses VI and obtains the auxiliary distribution through KL divergence minimizing and joint posterior density maximizing (we call joint distributed CE in the following). In 2019, Bellili et al. [21] proposed a novel Bayes-optimal channel estimation method that makes use of approximate belief propagation Bayesian networks. In 2017, Cheng et al. [28] proposed a different variational Bayesian inference-based channel estimation algorithm using a Gaussian mixture as a prior distribution. By using this Gaussian mixture in the prior distribution, the proposed algorithm can make the best use of sparsity within a single channel matrix, as well as between different channel matrices. In 2019, Thoota et al. [29] proposed another variational Bayesian channel estimation algorithm to estimate channel coefficients and the hyper-parameter of the transmitted symbol in MIMO cases. They modeled the MIMO system as a directed graphical model and so, despite the advantages offered by VI combined with data subsampling, i.e., providing approximate posterior inference over large data sets, their approach suffers from a poor local optimum (see Mandt et al. [30]). Some researchers use an annealing approach to tackle this problem, but annealing is only effective when choosing the appropriate cooling schedule (see Mandt et al. [30]).

To sidestep the disadvantages of the above methods of CE, we propose a variational channel estimation with tempering, and solve the problem of channel estimation in a MIMO scenario, and test it using simulations. Variational tempering (VT) is derived from Markov Chain Monte Carlo (MCMC) methods. By sampling from different temperatures, a MCMC method can increase the mixing time of the Markov chain [31]. In a similar way, we introduce global temperature. On the contrary, VT algorithms can learn a temperature distribution from the data, and update the weight in every iteration, which is similar to treating the transmitted signal of each sensor user as a distribution as well as its channel coefficiency. Additionally, we apply our CEVTI in coded CDMA case and uplink massive MIMO case and analyze its complexity and optimality guarantee. We find that CEVTI has better accuracy compared with state-of-the-art CE algorithms, satisfying computational cost and guarantees local optimality.

### 1.3. Objective

To overcome the problems of the algorithms discussed above, it is necessary to develop a new algorithm that is efficient, operational, and relatively simple. We use variational tempering to meet these requirements. In this work, we consider the channel estimation problem of the pilot signal and channel coefficients, assuming there is orthogonal access between the different sensors and the data fusion center. To reduce the complexity of the direct joint posterior density maximization, we propose a simpler VI algorithm that solves the channel estimation problem, adopting the concept of tempering. By formulating the channel estimation problem into a probabilistic graphical model, CEVTI can estimate the channel coefficiency and the transmitted signal in a low-complexity manner while simultaneously guaranteeing convergence at the same time. Simulations show that CEVTI has higher accuracy than state-of-the art algorithms.

The main objective of this paper is to obtain channel coefficiency by making the best use of trained pilot signals. To achieve this objective, we have performed the following tasks:

1. Modeling of the channel estimation problem into a variational message passing algorithm;
2. Evaluation of the performance error bound of our novel CEVTI algorithm;
3. Use of a Monte Carlo simulation to verify the bit error rate (BER), convergence rate, and mutual information of the CEVTI approach; and
4. Established the efficiency and superiority of our new algorithm, CEVTI.

### 1.4. Contributions

Over the past few years, VI has been explored in artificial intelligence, crowdsourcing and computer vision, etc. It has been proved an efficient way to compute hyper-parameters, and ground truth in various backgrounds that can be modeled as inference problems.

We propose a new channel estimation algorithm for MIMO-OFDM (orthogonal frequency division multiplexing) systems based on the VI approach. The CEVTI algorithm first models the channel estimation problem into a probabilistic graphical model, and then uses a factorized distribution to approximate the marginal distributions of the desired variables. Finally, the CEVTI algorithm solves this optimization problem in an alternative manner, just like the EM algorithm. Simulations show that CEVTI performs better than the state-of-the-art channel estimation algorithms.

This paper’s main contribution is the development of a new algorithmic approach to channel estimation in WSNs, as discussed below. Based on a variational message passing algorithm, a kind of probabilistic graphical model, this approach solves the channel estimation problem. First, we make an assumption about the user’s signal generative distribution, and then infer the channel coefficiency by using pilot feedback. Then we apply CEVTI in the MIMO-OFDM scenario and uplink massive MIMO scenario, and analyze its complexity and optimality. Analysis and simulations show that our algorithm can also be used in a higher dimension and results in better performance than the current channel estimation algorithms.

The main contributions of the paper can be summarized as follows:

1. The modeling of the OFDM channel estimation problem into a new variational message passing algorithm;
2. An evaluation of the performance error bound of our innovative variational tempering channel estimation algorithm; and
3. A numerical simulation of the performance of CEVTI to show that in general cases, the proposed CEVTI algorithm performs better than other algorithms.

In the rest of the paper, we first review the background of channel estimation model and variational inference in Section 2, and then define the background assumptions of the inference model in Section 3. Next, we derive our algorithm stepwise in Section 4, and show the application of CEVTI in Section 5. Next, we show how we obtain the results in Section 6, where we also compare the results of our algorithm with the performance of other approaches. Our CEVTI algorithm requires fewer constraints on the prior distributions of users and channels, and it performs better than the existing channel estimation algorithms. Section 7 presents the conclusion of this study.

## 2. Background

In our CEVTI system, we consider the case of CDMA, in which there are *N* antennas (which we term users in the rest of this paper) in the base station. At first, each user will send a signal to its antenna within communication range. The signal experiences relay block fading [32]. We assume that the transmitted signal of each antenna is uniformly distributed. Please note that the transmitted signal is first coded and preprocessed. The code rate of each user is $R_{c}$. We use $C_{i}$ to denote the codeword set of user *i* after interleaving. Each codeword then is multiplexed with pilot symbols, the number of which is $L_{p}$.

In this section, we describe the background notations of CEVTI, assuming that in the wireless sensor network, there are *M* mobile users and *N* antennas for each BS. We use $d_{i}$ to denote the transmitted symbol of mobile user *i*.

Suppose that each antenna has the state value of {±1}, and that we have defined $d_{i}$ to denote the transmitted symbol of user *i*, i.e., $d_{i}\in \left\{\pm 1\right\}$. Therefore, $d_{i}\left[l\right]$ is the *l*th transmitted signal, and column vector $d_{i}={\left[d_{i,p},d_{i,c}\right]}^{T}=\left[d_{i}\left[0\right],...,d_{i}\left[L-1\right]\right]$ is the symbol vector transmitted by user *i*. We use $d_{i,p}$ to denote the pilot symbol of user *i*, the length of which is $L_{p}$, and $d_{i,c}\in C_{k}$ to denote the codeword of user *i*, the length of which is $L_{c}$. Obviously $L=L_{c}+L_{p}$. For ease of reference, we treat both $d_{i}$ and $d_{i,c}$ as the codeword vectors of user *i*. The transmitted symbol of user *i* is then modulated by a normalized sequence embedding $s_{i}\left[l\right]$ of length $N_{c}$. Through a channel, the transmitted signal then experiences channel gain, including noise, fading, shifting, etc. However, we assume that the channel gain remains constant in one block time and differs in different blocks.

The vector $d_{i}\in C_{k}$ denotes the combination of the pilot symbol vector and the codeword vector; the length of the latter is $L_{c}$. Therefore, the dimension of $d_{i}$ is *L* (we may also refer to $d_{i}$ as a codeword to simplify notation). Every symbol is first processed by a signature waveform, and then is further altered by the transmitted channel, including channel gains, block fading etc. We assume the within each block, consisting of *L* symbols, the channel influence is constant, while between blocks, channel gains are different.

Next, we use $r\left[l\right]={\left[r_{1}\left[l\right],...,r_{N_{c}}\left[l\right]\right]}^{T}$ to indicate the receiving signal at interval *l*. So that the received signal after experiencing coding and channel gain is:

(1) $$\begin{array}{c} r[l]=S[l]\mathrm{Ad}[l]+w[l]=S[l]D[l]a+w[l] \end{array}$$

where S[l] is the spreading sequences of all users at signaling interval *l*, of which $a≜{\left[a_{1},...,a_{N}\right]}^{T}$ is the channel coefficient, and $A=\mathrm{diag}\{a_{1},...,a_{N}\}$ is the matrix of diagonalized channel coefficient, of which $a_{i}$ is the channel coefficient value of user *i*. Assuming a Rayleigh-fading channel and white Gaussian noise distribution, we have $a∼N(0,\Sigma_{a})$.

## 3. Solution Framework

Here, we introduce the CEVTI algorithm and then form its derivations. To consider a specific application of CEVTI, we apply it to the case of a coded multiuser system. In this multiuser scenario, we have multiple users whose channel coefficients must be calculated.

First, given the output of filters at signaling interval *l*r[l], channel coefficient vector a, and transmitted signal d, we obtain the joint probability of transmitted signals, channel coefficients, and output signal matrix, conditioned on the hyper-parameter of *a* α and the hyper-parameter of *d* θ:

(2) $$\begin{array}{cc} p(d,a,r|\alpha ,\theta ) & \;\;\propto \;\;\prod_{i\in [N]}\;\;p\left(a|\alpha \right)p\left(d|\theta \right)p\left(r|a,d\right) \end{array}$$

Please note that we assume channel coefficients, transmitted signals and output signals are independent with each other, for instance, $p\left(d|\theta \right)=\prod_{i\in [N]}\;\;p\left(d|\theta \right)$, and the joint probability of transmitted signals, channel coefficients, and output signal matrix can be extended using the chain rule in Bayesian network to simplify computation.

According to Bayesian theory, the estimation of *d* that has minimum error rate is:

(3) $$\begin{array}{cc} \hat{d_{i}} & =\arg\;\max_{d_{i}}p(d_{i}|r,\alpha )\;\\ \; & \mathrm{where}\;p\left(d_{i}|r,\alpha \right)=\sum_{i\in N_{j}\backslash i}\int_{a}p(a,d|r,\alpha )da \end{array}$$

of which $N_{j}$ is the neighbor of base station antenna *j*.

We can directly see that computing $p(d_{i}|\alpha ,\theta )$ is trivial since p(a,d|r,α) is unavailable. To solve this problem, we can borrow a concept from belief propagation (BP) and the mean-field method (MF) that uses a more factorable distribution to approximate the conditional probability in the above equation.

### 3.1. Mean-Field CEVTI

This subsection focuses on the hierarchical Bayesian model. In this model, all users share a global variable that determines the distribution of channel coefficients, while each local hidden variable belongs to each user. We denote $r=r_{1:N}$ as observed variables, $d=d_{1:N}$ as local hidden values, and α as global hidden values. Using a variational distribution q(a|α) to approximate p(a|α), and a variational distribution q(d|θ) to approximate p(d|θ), the joint density of the model, according to Equation (2), then becomes:

(4) $$p\left(a,r,d\right)=q\left(a|\alpha \right)q\left(d|\theta \right)\prod_{i=1}^{N}p\left(r_{i},d_{i}|\theta \right),$$

of which α is the global variable, i.e., the hyper-parameter of this model, and θ is the hidden local variable.

The main computing problem of the Bayesian model is the posterior inference, i.e., the output signal (or received signal). Our objective is to calculate p(a,d|r), the conditional probability of the hidden variable (the transmitted signal) and the global hidden variables (the hyper-parameter of channel coefficients) given the observations (received signal). In many cases, this computation is intractable, and approximation is required.

CEVTI proposes a parameterized family of hidden variables and tries to determine the family of members that are closest to the posterior distribution by KL divergence. That is to say, CEVTI tries to use the variational distribution to replace the target distribution. In VI, this is equal to computing the ELBO in terms of a variational parameter, since maximizing ELBO and minimizing KL divergence between the variational distribution and target distribution, are equivalent to maximizing the posterior probability:

(5) $$\begin{array}{cc} \log p(r) & =E_{q}\left[\log\left(\frac{p(a,d,r)}{q(a|\alpha )q(d|\theta )}\right)\right]-E_{q}\left[\log\left(\frac{p(a,d|r)}{q(a|\alpha )q(d|\theta )}\right)\right]\\ \; & =E_{q}\left[\log p\left(a,d,r\right)\right]-E_{q}\left[\log q\left(a,d|v\right)\right]-E_{q}\left[\log\left(\frac{p(a,d|r)}{q(a|\alpha )q(d|\theta )}\right)\right]\\ \; & =L(v)+KL(q(a,d)||p(a,d|r)) \end{array}$$

of which

(6) $$L\left(v\right)=E_{q}\left[\log p\left(a,d,r\right)\right]-E_{q}\left[\log q\left(a,d|v\right)\right]$$

Using the fully factored family, we assume that each user and its transmitted signals are independent

(7) $$q\left(d,a|v\right)=q\left(d|\lambda \right)\prod q\left(a_{i}|\phi_{i}\right)$$

where *v* is the variational parameter, λ is the global variational variable, $\phi_{i}$ is the local variational variable of user *i*, and therefore v={λ,ϕ}. This kind of simplification method is also called the mean-field method, and offers several computational advantages, especially when deriving the gradients of the objective function. VI optimizes Equation (6) using a gradient or coordinate ascent. To best determine the local optimal, we use tempered variational inference.

Tempered variational inference applies tempering into mean-field variational inference. We first use an additional temperature parameter T≥1. Given *T*, the joint probability becomes:

(8) $$p\left(d,a,r|T\right)=\frac{p{\left(d,r|a\right)}^{1/T}p\left(a|\alpha \right)}{c(T)}.$$

where c(T) is the normalizing constant, or the tempered partition function:

(9) $$c\left(T\right)=\int p{\left(d,r|a\right)}^{1/T}p\left(a|\alpha \right)drdadd.$$

The tempered joint means the tempered posterior. Tempered variational inference optimizes the variational distribution $q(\dot{)}$ against a tempered posterior. We start from a higher temperature and end when T=1. The tempered ELBO then becomes:

(10) $$\begin{array}{cc} L_{A}\left(\lambda ,\phi ;T\right) & =\;\;E_{q}\left[\log p\left(a|\alpha \;\right)\;\right]\;\;\;-\;\;E_{q}\left[\log q\left(a|\lambda \right)\right]\\ \; & +\sum_{i=1}^{N}\left(E_{q}\left[\log p\left(d_{i},r_{i}|a\right)\right]\right)/T-E_{q}\left[\log p\left(d_{i}|\phi_{i}\right)\right] \end{array}$$

We introduce a random variable that produces temperature to the joint distribution. We obtain the joint distribution of tempering at this temperature based on the results of random variables and use the following polynomial temperature distribution

(11) y∼Mult(π)

### 3.2. Tempered Joint

Using the chain rule of Bayesian theory, the joint distribution can be factorized as p(r,d,a,y)=p(r,d,a|y)p(y). We use uniform temperature distribution $p\left(y\right)=\prod_{m=1}^{M}\pi_{m}^{y_{m}}$. Conditioned on *y*, the tempered joint can be defined as:

(12) $$p\left(r,d,a|y\right)=\frac{p(\alpha )}{c(T_{y})}\prod_{i=1}^{N}p{\left(a,d_{i}|\alpha \right)}^{1/T_{y}}$$

Thus, the model becomes:

(13) $$p\left(r,a,d,y\right)=p\left(\alpha \right)\prod_{m=1}^{M}{\left(\frac{\pi_{m}}{c(T_{m})}\prod_{i=1}^{N}p{\left(a,d_{i}|\alpha \right)}^{1/T_{m}}\right)}^{y_{m}}$$

### 3.3. Tempered ELBO

Now we have defined the variational objective of the extended model. Next, we implement the mean-field family into a model containing temperature:

(14) p(d,a,y|ϕ,λ,γ)=q(d|ϕ)q(a|λ)q(y|γ)

in which γ is variational parameter for temperature. The tempered ELBO is:

(15) $$\begin{array}{cc} L_{T}\left(\lambda ,\phi ;T\right) & =E_{q}\left[\log p\left(d\right)\right]+E_{q}\left[\log\;p\left(a|\alpha \right)\right]+E_{q}\left[\log\;p\left(y\right)\right]\\ \; & -E_{q}\left[\log\;q\left(\theta \right)\right]\\ \; & -E_{q}\left[1/T_{y}\right]\sum_{i=1}^{N}\left(E_{q}\left[\log p\left(a,d_{i}|\alpha \right)\right]\right)\\ \; & -E_{q}\left[\log C\left(T_{y}\right)\right]-\sum_{i=1}^{N}E_{q}\left[\log q\left(d_{i}\right)\right]-E_{q}\left[\log q\left(y\right)\right] \end{array}$$

### 3.4. Local Variational

To make the calculation easier to process, we can use the local temperature for each user instead of the global temperature. This approach also makes it easier to process future data and to learn the tempering schedule of a single data. This local temperature can be viewed as the local energy potential across the network. We represent $t_{i}$ as the local temperature of each data, and the joint probability can be expressed as:

(16) $$\begin{array}{cc} p(r,d,a,t) & \propto p\left(a|\alpha \right)\prod_{i=1}^{N}\left[p{\left(r_{i},d_{i}|a\right)}^{1/t_{i}}p\left(t_{i}\right)\right] \end{array}$$

According to the above equation, the temperature can down-weight global hidden variables, making the local distribution more entropic. That is to say, at first, we put little weight on the influence of training data. In this way, we can quickly find a good initial result. In addition, then, we gradually optimize the result by putting more weight on the training data. Thanks to this effect, we will not have to calculate the tempered partition function, because the local tempering likelihood and the tempered partition function will be in the same family as the original prior of user distribution. The disadvantage; however, is that we cannot reach a conjugate model. Local temperature shows the likelihood of the data that comes from CEVTI without tempering. By assigning them higher temperatures, we can better explain outliers. In other words, local temperature enables us to model the data more flexibly, and to learn different tempering schedules for each data point during inference.

## 4. The CEVTI Algorithm

We now show how we derive our CEVTI algorithm. Our algorithm borrows from the concept of stochastic variational inference (see Hoffman et al. [33]), and combine it further with tempering. As in Hoffman et al., we focus on the conditional conjugate exponential family (CCEF). If in the model, the prior of user distribution and the local conditional probability belong to the same exponential family, and are conjugate, then the model is in the CCEF:

(17) $$\begin{array}{c} p\left(a|\alpha \right)=h\left(a\right)\exp\{\alpha^{T}t\left(a\right)-a_{g}\left(\theta \right)\} \end{array}$$

(18) $$\begin{array}{c} p\left(d_{i},r_{i}|a\right)=h\left(r_{i},d_{i}\right)\exp\left\{a^{T}t\left(d_{i},r_{i}\right)-a_{l}\left(a\right)\right\} \end{array}$$

where t(a) and $t(d_{i},r_{i})$ are sufficient statistics of global and local data points, and the sufficient statistics of *a* are $t\left(a\right)=(a,-a_{l}\left(a\right))$ (with slight abuse of notations, since *l* is the interval indicator of the transmitted signals, the subscripts *g* and *l* refer to global and local respectively). Therefore, hyper-parameter α consists of two components, $\left(\alpha_{1},\alpha_{2}\right)$, the former is a vector that has the same dimension as *a*, and the latter is a scalar vector. Notice that the conditional probability of the global variable is in the same exponential family as the prior of natural parameter $\eta_{g}$, of which $a_{g}\left(\theta \right)$ and $a_{l}\left(d\right)$ are the corresponding log normalizers (here *a* is different from channel coefficients a). We choose CCEF because it allows us to compute expectations analytically.

We treat the following objective function as a function of the global variational parameter

(19) $$\begin{array}{c} L_{T}\left(\lambda ,\phi ;T\right)=L_{T}\left(\lambda ,\phi_{ij};T\right) \end{array}$$

(20) $$\begin{array}{c} \phi \left(\lambda ,T\right)=\arg\;\max_{\phi_{ij}}L_{T}\left(\lambda ,\phi_{ij};T\right) \end{array}$$

According to Hoffman et al. [33], the ELBO after combining with tempering is:

(21) $$\begin{array}{cc} L_{T}\left(\lambda ,\phi ;T\right) & =E_{q}{\left[1/T_{y}\eta_{g}\left(r,d,\alpha \right)\right]}^{T}\nabla_{\lambda }a_{g}\left(\lambda \right)-\lambda^{T}\nabla_{\lambda }a_{g}\left(\lambda \right)\\ \; & +a_{g}\left(\lambda \right)-E_{q}\left[\log C\left(T_{y}\right)\right]-E_{q}\left[\log q\left(y\right)\right] \end{array}$$

of which

(22) $$\begin{array}{c} \eta_{g}\left(r,d,\alpha \right)=\left(\alpha_{1}+\sum_{i=1}^{N}t\left(r_{i},d_{i}\right),\alpha_{2}+N\right) \end{array}$$

### Updates

According to Hoffman et al. [33], the tempered ELBO’s natural gradient with respect to the global variational parameter is:

(23) $$\begin{array}{c} \nabla_{\lambda }\phi \left(\lambda ,T\right)=E_{q}\left[\eta_{g}\left(r,d,\alpha \right)\right]-\lambda \end{array}$$

(24) $$\begin{array}{c} \hat{\lambda }=E_{q}\left[\eta_{g}\left(r,d,\alpha \right)\right] \end{array}$$

(25) $$\begin{array}{c} \lambda_{t+1}=\lambda_{t}+\rho \left(\hat{\lambda }-\lambda_{t}\right) \end{array}$$

In the first step, we estimate $\hat{\lambda }$, and then update it using $\lambda_{t}$ with decreasing learning rate $\rho_{t}$. By dividing the expectation of the sufficient statistics, we can reduce the variance of the gradients.

Then we optimize the tempered ELBO, so that the local variational update is:

(26) $$\begin{array}{c} \phi_{ij}=\frac{1}{T}E_{q}\left[\eta_{l}\left(t_{i,-j},a_{i},\theta \right)\right] \end{array}$$

of which $\eta_{l}$ is local variational parameter.

## 5. Application of CEVTI

In this section, we apply the CEVTI algorithm to the case of coded Code Division Multiple Access (CDMA) and in the case of uplink massive MIMO systems. As discussed above, CEVTI works as a formal optimization framework based on variational inference with tempering. It has the properties of guaranteed convergence and smooth descent, which lead to a better local optimum.

### 5.1. Application of CEVTI for CDMA

Assuming that the prior distribution of a is Gaussian, i.e.,

(27) $$\begin{array}{c} p(a)\;\propto \;\exp\{-a^{H}\Sigma_{a}a\} \end{array}$$

we can rewrite the CEVTI objective as:

(28) $$\begin{array}{cc} \mathrm{Maximize}L_{T}\left(\lambda ,\phi ;T\right) & =E_{q}{\left[1/T_{y}\eta_{g}\left(r,d,\alpha \right)\right]}^{T}\nabla_{\lambda }a_{g}\left(\lambda \right)\\ \; & -\lambda^{T}\nabla_{\lambda }a_{g}\left(\lambda \right)\\ \; & +a_{g}\left(\lambda \right)-E_{q}\left[\log C\left(T_{y}\right)\right]-E_{q}\left[\log q\left(y\right)\right]\\ \mathrm{subject}\;\mathrm{to} & \int_{a}da=1\\ \; & \sum_{i}^{N}d_{i}=1 \end{array}$$

#### 5.1.1. Channel Coefficient Estimation

According to Equation (26), the update of the channel coefficient vector is:

(29) $$\begin{array}{c} q_{a}^{t+1}\left(a\right)\propto \frac{1}{T}\exp\sum_{d_{i}}q_{d_{i}}^{t}\eta_{g}\left(r,d,\alpha \right) \end{array}$$

Similarly, the update of the codeword parameter is:

(30) $$\begin{array}{c} q_{d_{i}}^{t+1}\left(d_{i}\right)\propto \frac{1}{T}\exp\int da\sum_{d_{-i}}q_{d_{-i}}^{t}\eta_{l}\left(r,d,\alpha \right) \end{array}$$

Observe that when $d_{i}\notin C_{i}$, $q_{d_{i}}=0$. Furthermore, $\eta_{g}\left(r,d,\alpha \right)\propto \log p\left(a,d|r\right)$, which is not intuitive to compute, it can be simplified by computing p(r|a,d) instead, since by the chain rule, p(a,d|textbfr)=p(r|a,d)p(a)p(d)/p(r), and dropping p(d) when updating p(a) does not impact the result. According to Equation (1), we can easily compute the analytical distribution of p(r|a,d) using Gaussian density equation. Therefore, the above equation can be rewritten as:

(31) $$\begin{array}{c} q_{a}^{t+1}\left(a\right)\propto \frac{1}{T}\exp\sum_{d_{i}}q_{d_{i}}^{t}\log p\left(r|a,d\right) \end{array}$$

Similarly, the update of the codeword parameter is:

(32) $$\begin{array}{c} q_{d_{i}}^{t+1}\left(d_{i}\right)\propto \frac{1}{T}\exp\int da\sum_{d_{-i}}q_{d_{-i}}^{t}\log p\left(r|a,d\right) \end{array}$$

Assuming the noise vector w[l] of Equation (1) is $\Sigma_{w}$, and a is also Gaussian distribution, we need to update the mean and covariance of a separately, i.e.,

(33) $$\begin{array}{c} q_{a}^{t+1}\left(a\right)\propto \frac{1}{T}\exp\sum_{d_{i}}q_{d_{i}}^{t}\log p\left(r|a,d\right) \end{array}$$

in which

(34) $$\begin{array}{c} \log p\left(r|a,d,\Sigma_{w}\right)=\mathrm{const}*{\left|\Sigma_{w}\right|}^{-1}\\ \exp\left(r\left[l\right]-S\left[l\right]D\left[l\right]a\right)\Sigma_{w}^{-1}{\left(r\left[l\right]-S\left[l\right]D\left[l\right]a\right)}^{H} \end{array}$$

Defining

(35) $$\begin{array}{c} d_{i}^{t}\left[l\right]≜E_{q_{d}^{t}}\left\{d_{i}\left[l\right]\right\}=E_{q_{d_{i}}^{t}}\left\{d_{i}\left[l\right]\right\}\\ =\sum_{d_{i}\in C_{i},d_{i}\left[l\right]=1}q_{d_{i}}^{t}\left(d_{i}\right)-\sum_{d_{i}\in C_{i},d_{i}\left[l\right]=-1}q_{d_{i}}^{t}\left(d_{i}\right) \end{array}$$

(36) $$E_{q_{d}^{t}}\left\{d_{i}\left[l\right]*d_{j}\left[l\right]\right\}≜\left\{\begin{array}{cc} d_{i}\left[l\right]*d_{j}\left[l\right], & i\neq j,\\ 1, & i=j \end{array}\right.$$

and

(37) $$\begin{array}{c} {\left(\Omega_{w}^{t}\right)}^{-1}≜E_{q_{\Sigma_{w}^{-1}}^{t}}\left\{\Sigma_{w}^{-1}\right\} \end{array}$$

so that:

(38) $$\begin{array}{c} q_{a}^{t+1}\left(a\right)\propto \frac{1}{T}\exp\left[-{\left(a-a^{t+1}\right)}^{H}{\Sigma_{a}^{t+1}}^{-1}\left(a-a^{t+1}\right)\right] \end{array}$$

where a is:

(39) $$\begin{array}{cc} a_{\mathrm{mean}}^{t+1} & =\Sigma_{a}^{t+1}(\sum_{l=0}^{L_{p}-1}D_{p}{\left[l\right]}^{H}S{\left[l\right]}^{H}{\left(\Omega_{w}^{t}\right)}^{-1}r\left[l\right]\\ \; & +\sum_{l=L_{p}}^{L-1}{\tilde{D}}^{t}{\left[l\right]}^{H}S{\left[l\right]}^{H}{\left(\Omega_{w}^{t}\right)}^{-1}r\left[l\right]) \end{array}$$

and the covariance of a is:

(40) $$\begin{array}{cc} \Sigma_{a}^{t+1} & =\frac{1}{T^{2}}(\Sigma_{a}^{-1}+\sum_{l=0}^{L_{p}-1}D_{p}{\left[l\right]}^{H}S{\left[l\right]}^{H}{\left(\Omega_{w}^{t}\right)}^{-1}S\left[l\right]D_{p}\left[l\right]\\ \; & +\sum_{l=L_{p}}^{L-1}{\tilde{D}}^{t}{\left[l\right]}^{H}S{\left[l\right]}^{H}{\left(\Omega_{w}^{t}\right)}^{-1}S\left[l\right]\tilde{D}\left[l\right]\\ \; & +\sum_{l=L_{p}}^{L-1}E^{t}{\left[l\right]}^{H}\mathrm{Diag}\left\{S{\left[l\right]}^{H}{\left(\Omega_{w}^{t}\right)}^{-1}S\left[l\right]\right\}E^{t}\left[l\right]{)}^{-1} \end{array}$$

in which $E^{t}=\mathrm{diag}\left\{1-{\left({\tilde{d}}_{1}{\left[l\right]}^{t}\right)}^{2},\cdots ,1-{\left({\tilde{d}}_{N}{\left[l\right]}^{t}\right)}^{2}\right\}$.

#### 5.1.2. Noise Covariance Estimation

Here we estimate the noise covariance matrix of our CDMA channel estimation model. Notice that in the original derivation of CEVTI, we do not include $\Sigma_{w}^{-1}$ as the targeted parameter to be estimated, because CEVTI can be extended to multiple dimensions flexibly, without complex modification.

Similarly, we reach the update equation of noise covariance as follows:

(41) $$\begin{array}{c} q_{\Sigma_{w}^{-1}}^{t+1}\left(\Sigma_{w}^{-1}\right)\propto \frac{1}{T}p\left(\Sigma_{w}^{-1}\right)\exp\int_{a}da\sum_{d_{i}}q_{d_{i}}^{t}\log p\left(r|a,d\right) \end{array}$$

Defining

(42) $$\begin{array}{c} B_{t}≜\exp\int_{a}da\sum_{d_{i}}q_{d_{i}}^{t}\log p\left(r|a,d\right)\\ =\sum_{l=0}^{L_{p}-1}(\left(r\left[l\right]-S\left[l\right]D_{p}\left[l\right]a^{t}\right){\left(r\left[l\right]-S\left[l\right]D_{p}\left[l\right]a^{t}\right)}^{H}\\ +S\left[l\right]D_{p}\left[l\right]\Sigma_{a}^{t}{D_{p}}^{t}{\left[l\right]}^{H}S{\left[l\right]}^{H})\\ +\sum_{l=L_{p}}^{L-1}(\left(r\left[l\right]-S\left[l\right]{\tilde{D}}^{t}\left[l\right]a^{t}\right){\left(r\left[l\right]-S\left[l\right]{\tilde{D}}^{t}\left[l\right]a^{t}\right)}^{H}\\ +S\left[l\right]E^{t}\left[l\right]A^{t}{\left(A^{t}\right)}^{H}{\left(E^{t}\left[l\right]\right)}^{H}\left(S{\left[l\right]}^{H}\right)\\ +S\left[l\right]E^{t}\left[l\right]\mathrm{Diag}\left\{\Sigma_{a}^{t}\right\}{\left(E^{t}\left[l\right]\right)}^{H}\left(S{\left[l\right]}^{H}\right)\\ +S\left[l\right]{\tilde{D}}^{t}\left[l\right]\Sigma_{a}^{t}{{\tilde{D}}^{t}\left[l\right]}^{H}S{\left[l\right]}^{H}) \end{array}$$

(43) $$\begin{array}{c} q_{\Sigma_{w}^{-1}}^{t+1}\left(\Sigma_{w}^{-1}\right)\propto \exp\int_{a}da\sum_{d_{i}}q_{d_{i}}^{t}{\left|\Sigma_{w}^{-1}\right|}^{L}\exp\left[-tr\left\{\Sigma_{w}^{-1}B^{t}\right\}\right] \end{array}$$

Therefore, $\Sigma_{w}^{-1}$ is a Wishart distribution, whose expectation is the multiplication of dimension and the inverse covariance matrix, which is

(44) $$\begin{array}{c} {\left(\Omega_{w}^{t}\right)}^{-1}=\frac{L+N+1}{B^{t}} \end{array}$$

#### 5.1.3. Codeword Distribution Estimation

We estimate the codeword distribution matrix of our CDMA channel estimation model in this subsection.

Similar to the noise covariance estimation, we obtain the updated equation of the codeword distribution as follows:

(45) $$\begin{array}{c} q_{{d_{i}}^{t+1}}\left({d_{i}}^{t+1}\right)\propto \frac{1}{T}p\left(d_{i}\right)\exp\int_{a}da\sum_{d_{i}}q_{d_{i}}^{t}\log p\left(r|a,d\right) \end{array}$$

Assuming that the distribution of the codeword is a uniform distribution, and defining:

(46) $$\begin{array}{c} C_{i}^{t+1}\left[l\right]\;\;\;\;≜\exp\int_{\Sigma_{w}^{-1}}d\Sigma_{w}^{-1}\;\;\int_{a}da\sum_{d_{i}}\;\;q_{d_{i}}^{t}\log p\left(r|a,d\right)\\ \propto \exp\{-tr{\left(\Omega_{w}^{t}\right)}^{-1}\sum_{l=0}^{L_{p}-1}\left(r\left[l\right]-S\left[l\right]{\tilde{D}}_{i}\left[l\right]a^{t}\right){\left(r\left[l\right]-S\left[l\right]{\tilde{D}}_{i}\left[l\right]a^{t}\right)}^{H}\\ +S\left[l\right]{\tilde{D}}_{i}^{t}\left[l\right]\Sigma_{a}^{t}{{\tilde{D}}_{i}^{t}\left[l\right]}^{H}S{\left[l\right]}^{H}\} \end{array}$$

where ${\tilde{D}}_{i}^{t}\left[l\right]=\mathrm{diag}\left\{\ldots {\tilde{d}}_{i-1}^{t}\left[l\right]d_{i}^{t}\left[l\right]{\tilde{d}}_{i+1}^{t}\left[l\right]\ldots \right\}$. We can get:

(47) $$\begin{array}{c} q_{{d_{i}}^{t+1}}\left({d_{i}}^{t+1}\right)\propto \exp\left\{\sum_{l=0}^{L_{p}-1}d_{i}\left[l\right]C_{i}^{t+1}\left[l\right]\right\} \end{array}$$

### 5.2. Application of CEVTI in Massive MIMO

In this section, we apply the CEVTI algorithm to the case of uplink massive MIMO systems with low resolution ADCs. Similar to the above case in CDMA, in uplink massive MIMO systems with low resolution ADCs, base stations will quantize the received signals before decoding them, i.e.

(48) $$\begin{array}{c} Y=Q(r) \end{array}$$

of which r is the received signal before quantization (or modulation), Y is the received signal after quantization, and Q is the quantization operator. Since the antennas of massive MIMO systems use M-Quadrature Amplitude Modulation (M-QAM) to modulate the received signals, we need to transform the received signals with imaginary parts and real parts into real number. Therefore, the joint probability of transmitted signals, received signals before modulation, received signals after modulation, and channel coefficients are

(49) $$\begin{array}{cc} p(r,d,a,Y,t) & \propto p\left(a|\alpha \right)\prod_{i=1}^{N}\left[p\left(Y|r_{i}\right)p{\left(r_{i},d_{i}|a\right)}^{1/t_{i}}p\left(t_{i}\right)\right] \end{array}$$

Assume the noise is Gaussian distributed, the conditional distribution of the signals and channel coefficients are given as

(50) $$\begin{array}{cc} p(r|d,a) & \propto \exp(-\frac{1}{\sigma_{w}^{2}}\sum_{l=0}^{L-1}||r_{i}\left[l\right]-ad_{i}\left[l\right]|{|}^{2}) \end{array}$$

(51) $$\begin{array}{cc} p(a|\alpha ) & \propto \exp(-\sum_{j=0}^{M}\frac{1}{\alpha_{j}}||a_{j}|{|}^{2}) \end{array}$$

(52) $$\begin{array}{c} p\left(Y|d\right)=1(d_{i}\in \left[d_{i}^{lo},d_{i}^{hi}\right]) \end{array}$$

of which $\left[d_{i}^{lo},d_{i}^{hi}\right]$ are soft bounds of $d_{i}$.

Then, according to Equation (14), we can obtain the variational joint containing temperature:

(53) p(d,Y,a,y|ϕ,ζ,λ,γ)=q(d|ϕ)q(Y|ζ)q(a|λ)q(y|γ)

Therefore, the tempered ELBO is:

(54) $$\begin{array}{cc} L_{T}\left(\lambda ,\phi ;T\right) & =E_{q}\left[\log p\left(d\right)\right]+E_{q}\left[\log p\left(Y\right)\right]+E_{q}\left[\log\;p\left(a|\alpha \right)\right]+E_{q}\left[\log\;p\left(y\right)\right]\\ \; & -E_{q}\left[\log\;q\left(\theta \right)\right]\\ \; & -E_{q}\left[1/T_{y}\right]\sum_{i=1}^{N}\left(E_{q}\left[\log p\left(a,d_{i}|\alpha \right)\right]\right)\\ \; & -E_{q}\left[\log C\left(T_{y}\right)\right]-\sum_{i=1}^{N}E_{q}\left[\log q\left(d_{i}\right)\right]-\sum_{i=1}^{N}E_{q}\left[\log q\left(Y_{i}\right)\right]-E_{q}\left[\log q\left(y\right)\right] \end{array}$$

According to Equation (26), the update of the channel coefficient vector is:

(55) $$\begin{array}{c} a_{\mathrm{mean}}^{t+1}=\sigma_{w}^{t+1}\left(\sum_{l=0}^{L-1}D_{i}{\left[l\right]}^{H}{\left(\sigma_{w}^{t}\right)}^{-1}Y\left[l\right]\right) \end{array}$$

and the covariance of a is:

(56) $$\begin{array}{c} \sigma_{a}^{t+1}=\frac{\sigma_{w}^{2}}{\sum_{l=0}^{L-1}D_{i}^{t}\left[l\right]+\frac{\sigma_{w}^{2}}{\alpha_{j}}} \end{array}$$

And similarly, we reach the solution of $d_{i}\left[l\right]$ and $r_{i}\left[l\right]$:

(57) $$\begin{array}{c} q\left(d_{i}\left[l\right]=m\right)=\frac{\exp(-\frac{1}{\sigma_{w}^{2}}f\left(m\right))}{\sum_{m^{\prime }}\exp\left(-\frac{1}{\sigma_{w}^{2}}f\left(m^{\prime }\right)\right)} \end{array}$$

of which *m* is the value in M-QAM space, and

(58) $$\begin{array}{c} f\left(m\right)=E_{q}[∥a_{j}{∥}^{2}{]|m|}^{2}+2R\left[\left(\sum_{j^{\prime }=1,j^{\prime }\neq j}^{M}a_{j^{\prime }}^{H}a_{j}d_{i}^{j^{\prime }}{\left[l\right]}^{*}-r{\left[l\right]}^{H}a_{j}\right)m\right] \end{array}$$

(59) $$\begin{array}{c} r_{i}^{mean}\left[l\right]=E_{q}\left[ad_{i}\left[l\right]\right] \end{array}$$

(60) $$\begin{array}{c} r_{i}\left[l\right]∼N\left(E_{q}\left[ad_{i}\left[l\right]\right],\frac{\sigma_{w}}{\sqrt{2}}\right) \end{array}$$

## 6. Simulations and Results

In this section, we will numerically assess the performance of the five most popular channel estimation algorithms for the simulations in which we test the CEVTI algorithm. After comparing their performances, we analyze the CEVTI algorithm’s complexity and optimality guarantee.

### 6.1. Simulations

We perform Monte Carlo simulations in a typical communication system, and assume that the signal generating parameter is uniformly distributed as well as the channel gain. Some of these parameters are listed in Table 1.

We use the BER, convergence rate, and mutual information to show the accuracy of our CEVTI. In our pilot scheme, all transmitters transmit at the same time frequency and experience the same Rayleigh-fading channel. Furthermore, we assume there is no correlation between the transmitting antennas and the receiving antennas.

We compare CEVTI with five channel estimation algorithms: LS, LLMSE, joint distributed CE, CE for FDD, LDAMP and VMP. All the variational-based algorithms use the same initialization, i.e., a pilot-based channel estimation and a MIMO channel model.

**Figure 1:**
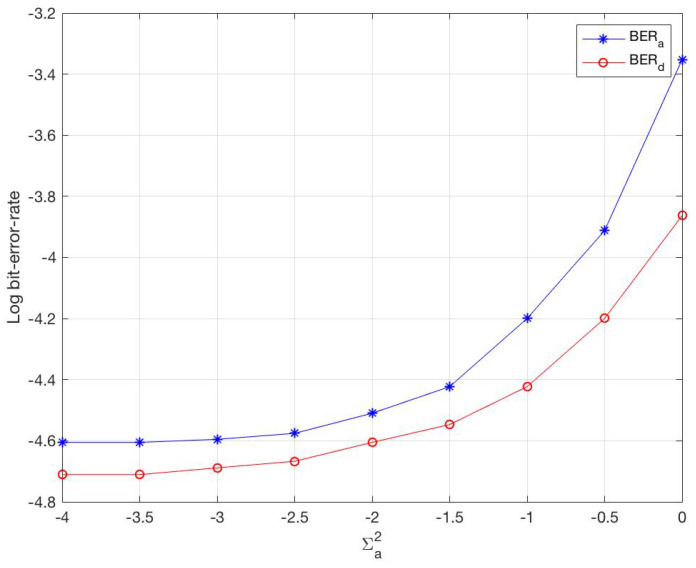
Noise Variance Impact.

Figure 1 shows the impact of noise variance on the estimation of the channel coefficient. It is intuitive that the BER increases with the noise variance at an acceleration rate. Please note that the sensor noise variance also has a huge impact on the estimation accuracy, and is more difficult to correct using CE algorithms, unless very large data samples are used. However, CEVTI displays higher accuracy compared with others, which means that CEVTI guarantees better local optimum.

**Figure 2:**
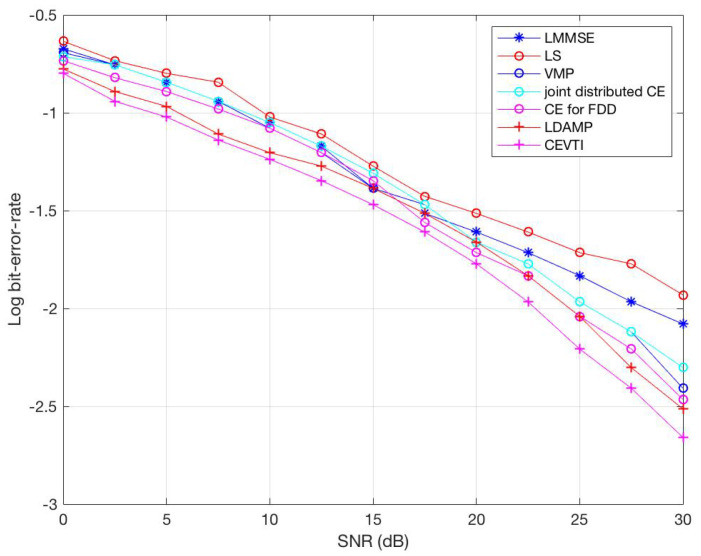
BER comparison.

Figure 2 shows the BER of the signal-to-noise ratio (SNR, i.e., $E_{b}/N_{0}$). We iterate 10 times to simplify the comparison. The results show that CEVTI has a better transmitter performance than the five other existing CE algorithms. The BER of CEVTI is significantly lower than LLMSE-based algorithms. In addition, the convergence rate of CEVTI is non-degenerate compared to other iterative CE algorithms. We can see that LDAMP also works well due to its computational power and use of large training data. The CEVTI algorithm is also expected to produce a local minimum BER, as it can make the best use of local data samples, even when the sample size is small.

**Figure 3:**
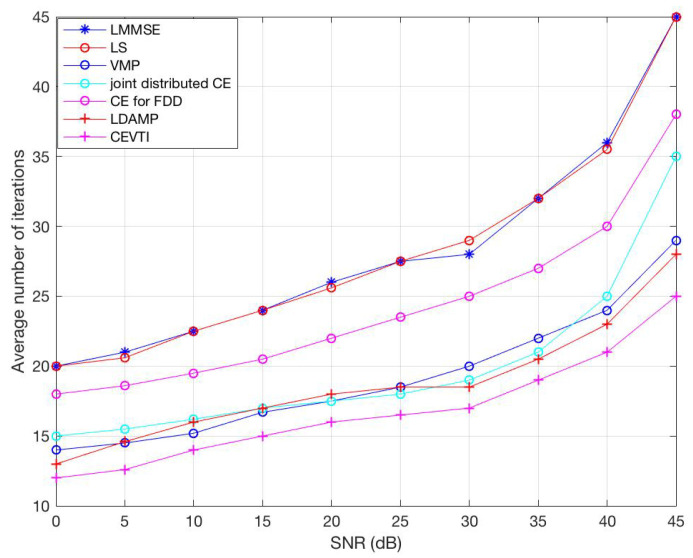
Average number of iterations until convergence.

Figure 3 shows the convergence behavior of CEVTI compared with LS, LLMSE, joint distributed CE, CE for FDD, LDAMP and VMP under different SNR. The results show that CEVTI converges faster and smoother than other CE algorithms. The reason is, as explained before, VT has guaranteed convergence and smooth descent. We can see from Figure 3, joint distributed CE and VMP also behave well due to the application of VI. For instance, at SNR=15dB, CEVTI converges in around 15 iterations, and joint distributed CE and VMP converge in 17 and 16 iterations, respectively. The simulated executing time of CEVTI is also better, showing that CEVTI has satisfying computational complexity.

**Figure 4:**
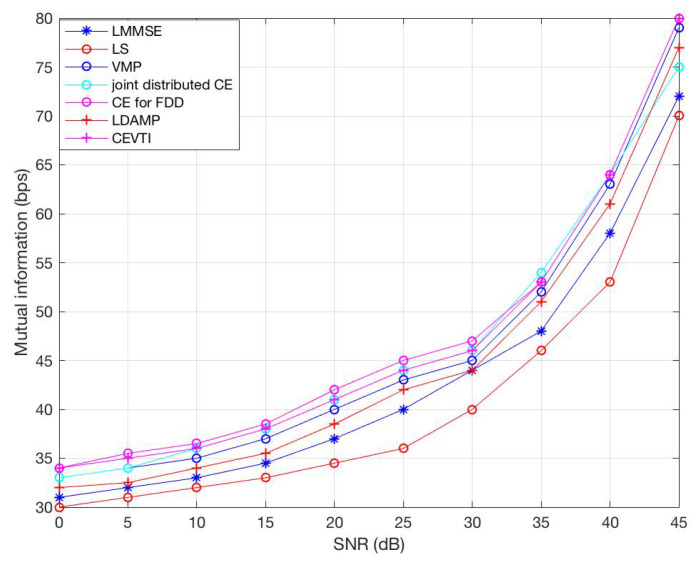
Mutual information comparison.

Figure 4 shows the mutual information comparison with LS, LLMSE, joint distributed CE, CE for FDD, LDAMP and VMP under different SNR. The mutual information is between users and BS antennas, which is a good indicator of the impact of channel estimation accuracy on the throughput of the average bandwidth. The results show that CEVTI has non-degenerate throughput compared with others. For instance, at SNR=20 dB, CEVTI’s mutual information is 41, same as VMP and joint CE, slightly higher than others, showing that CEVTI has theoretical satisfying network throughput.

### 6.2. Complexity Analysis

Here, we analyze the computational complexity of CEVTI. Since the main computing burden of CEVTI is the matrix inverse operation, we can only consider Equations (38)–(40). Since a N×N matrix takes $N^{3}$ calculations to calculate its inverse, we can see that Equation (38) takes $O(N^{3})$ calculations, and Equations (39) and (40) takes $O(L_{p}^{3}+{\left(L-L_{p}\right)}^{3})$ calculations. Therefore, the total complexity of CEVTI is $O(N^{3}+L_{p}^{3}+{\left(L-L_{p}\right)}^{3})$.

### 6.3. Optimality Guarantee

We now prove how our CEVTI has the local optimum guarantee.

**Theorem** **1** (Optimality Guarantee)**.** *Our CEVTI has optimality guarantee.*

**Proof** **of** **Theorem** **1.** Recall that the optimum of a constrained optimization problem, also called stationary point, or sometimes fixed point, has a set of gradients that is orthogonal to the constraint set [34]. Therefore, we need to prove that the result of CEVTI is a stationary point.

We first redefine our factor graph as a clique tree, with a set of vertex v={Musers,Nantennas}, and a set of edges e={useri−antennaj}. Please note that any graph that has a maximum clique size of 1 can also be treated with a clique tree. In this case, our factor graph is a clique tree with clique size 1. According to Koller et al. [35], the configuration q of a stationary point of a clique tree only exists when its energy potential has the following form:

(61) $$\begin{array}{c} f_{e_{i-j}}\propto \sum_{v_{i}-e_{i-j}}f_{v_{i}}\left(\prod_{k\in Nb_{i}-j}f_{e_{k-i}}\right) \end{array}$$

(62) $$\begin{array}{c} b_{i}\propto f_{v_{i}}\left(\prod_{j\in Nb_{i}-j}f_{e_{j-i}}\right) \end{array}$$

in which *f* is the energy of the vertex and the edges, and *b* is the belief of the vertex. Recall that Equation (33) can be rewritten as:

(63) $$\begin{array}{c} ln\left(q_{a}^{t+1}\left(a\right)\right)\propto \sum_{d_{i}}q_{d_{i}}^{t}\log p\left(r|a,d\right)\\ \propto \sum_{d_{i}}q_{d_{i}}^{t}\log p\left(a,d,r\right)\\ \propto \sum_{d_{i}}q_{d_{i}}^{t}\log p\left(a\right)\log p\left(d\right)\log p\left(r\right), \end{array}$$

which is exactly the form of Equation (61) if we treat a, d, r as the vertex, and the link between them as the edges. Therefore, the solution of our CEVTI is the stationary point of the variational optimization. □

## 7. Conclusions and Future Directions

To overcome the problems with the existing algorithms in channel state inference in WSNs, such as high complexity, poor generalization, impracticability and so on, we develop a new channel estimation method with variational tempering for MIMO-OFDM scenario. A ground truth information extraction algorithm based on variational tempering is proposed and implemented. Our CEVTI provides insights that account for multiple factors in inferring truth and can generalize to higher dimensions and finds better local optimum. As a variation of SVI, CEVTI can find out the optimal hyper-parameters of channels with fast convergence rate, and can be applied to the case of CDMA and uplink massive MIMO easily. As can be seen in Section 5, CEVTI can iteratively minimize the objective function with multiple dimensions. We demonstrate the performance of CEVTI through numerical simulation. The BER, convergence rate, and mutual information comparisons with the five existing CE algorithms show that CEVTI outperforms others under different noise variance and signal-to-noise ratio. Furthermore, the results show that the more parameters that are considered in each iteration, the faster the convergence rate and the lower the non-degenerate bit error rate with CEVTI. Analysis shows that CEVTI has satisfying computational complexity, and guarantees better local optimum. Therefore, this paper has contributed to the quest for developing efficient algorithms in artificial advanced sensor networks. Possible future research directions include investigation of how the graph structure can impact the performance of CEVTI, and constructing inference algorithms that can suit more complex situations. 

## Figures and Tables

**Table 1:** Parameter notations and values.

Parameter	Meaning	Value
$L_{p}$	pilot number	32
*N*	number of users	8
*M*	number of antennas	4
$r_{ij}$	receiver *j*’s signal toward user *i*	
$\left[\alpha_{1},\beta_{1}\right]$	signal prior	[0.5,1]
$\left[\alpha_{2},\beta_{2}\right]$	user prior	[0.4,0.6]
ϵ	convergence tolerance	$10^{-6}$

## Abbreviations

The following abbreviations are used in this manuscript:

WSNs	Wireless Sensor Networks
VI	Variational Inference
CEVTI	Channel Estimation Variational Tempering Inference
CE	Channel Estimation
CS	Compressive Sensing
CSI	Channel State Information
MP	Message Passing
ELBO	Evidence Lower Bound
MCMC	Monte Carlo Markov Chain
